# Adrenal Insufficiency During Treatment With Immune‐Checkpoint Inhibitors: How to Simplify the Diagnostic Pathway?

**DOI:** 10.1111/cen.70023

**Published:** 2025-08-28

**Authors:** Alice Nervo, Giovanni Gruosso, Marta Marino, Enrica Migliore, Matteo Rubiolo, Elisa Vaccaro, Emanuela Arvat

**Affiliations:** ^1^ Oncological Endocrinology Unit, AOU Città della Salute e della Scienza University of Turin Turin Italy; ^2^ Department of Clinical and Experimental Medicine University of Catania Catania Italy; ^3^ Cancer Epidemiology Unit University of Turin Turin Italy

**Keywords:** hypothalamic–pituitary–adrenal axis, immunotherapy, isolated ACTH deficiency, salivary cortisol, salivary cortisone, serum cortisol

## Abstract

**Objective and Background:**

Early identification of adrenal insufficiency (AI) during immune‐checkpoint inhibitors (ICIs) is crucial to prevent life‐threatening consequences; however, the diagnosis is challenging.

**Design, Patients and Measurement:**

In this prospective observational study, ICI‐treated cancer patients with morning serum cortisol (SC) within the grey zone (83–414 nmol/L) were enroled. A low‐dose adrenocorticotropic hormone (ACTH) stimulation test was performed to assess peak stimulated SC, preceded by re‐evaluation of morning SC. The analysis aimed to refine morning SC cut‐offs to minimize the diagnostic grey zone reducing the need for ACTH stimulation. To interpret the results of ACTH test, it was employed both the threshold for peak stimulated SC provided by the Endocrine Society guidelines (500 nmol/L) and an assay‐specific cut‐off for the Abbott platform recently proposed in literature (414 nmol/L). Moreover, the utility of salivary cortisol and cortisone upon awakening was explored.

**Results:**

In 30 ICI‐treated patients, a good positive correlation between morning and peak stimulated SC was confirmed (*r* = 0.72, *p* < 0.001); peak stimulated SC ≤ 500 nmol/L and ≤ 414 nmol/L was detected in 36.7% and 20% of subjects, respectively; all cases were secondary AI. ROC curve analysis identified optimal morning SC thresholds for AI prediction and exclusion (> 196 nmol/L and ≤ 397 nmol/L in case of pathological peak stimulated SC ≤ 500 nmol/L; > 163 nmol/L and ≤ 251 nmol/L in case of pathological peak stimulated SC ≤ 414 nmol/L). Salivary cortisol and cortisone upon awakening positively correlated with peak stimulated SC (*r* = 0.49 and *r* = 0.56, respectively, *p* < 0.01).

**Conclusions:**

Morning SC is a reliable tool for the screening of ICI‐related AI; the need for dynamic stimulation would be reduced by the application of assay‐specific cut‐offs. Salivary hormone analysis of cortisol and cortisone upon awakening may represent a viable alternative in selected cases.

## 1 Introduction

Immune‐checkpoint inhibitors (ICIs) targeting cytotoxic T‐lymphocyte‐associated protein‐4 (CTLA‐4), programmed‐death receptor‐1 (PD‐1) or its ligand (PD‐L1) have been successfully employed for the treatment of several malignancies [1].

ICI‐related adrenal insufficiency (AI) can be mostly due to immune‐related pituitary damage [2, 3]. Hypophysitis may occur mainly during anti CTLA‐4 treatment, with mass‐effect symptoms and/or pituitary enlargement, possibly accompanied by hypopituitarism. On PD‐1 and PD‐L1 inhibitors, an isolated adrenocorticotropic hormone (ACTH) deficiency (IAD) is more frequently detected [4, 5]. Rarely, an immune‐mediated damage may directly involve the adrenal cortex, with depletion of both glucocorticoids and mineralocorticoids [6].

The downstream effects of a hypothalamic–pituitary–adrenal (HPA) axis disruption might be acutely felt since serum cortisol (SC) has a short half‐life. In absence of an adequate hormonal replacement therapy, a life‐threatening adrenal crisis might occur, especially in case of stressful situations that are common in cancer patients.

However, early recognition of ICI‐related AI is challenging, and diagnosis cannot be easily performed in most cases. First, the initial presentation of AI might be insidious and characterized by non‐specific symptoms (e.g., asthenia, gastrointestinal disturbances, hypotension), frequently observed in oncologic patients on systemic treatments [7]. Since the clinical evaluation is often insufficient or misleading, a routinary biochemical screening for AI is recommended in all ICI‐treated patients [6, 8, 9]. Moreover, exogenous steroids are frequently used to treat non‐endocrine ICI‐related toxicity, therefore, current or recent glucocorticoid treatment might represent a possible confounder in the diagnostic process [10].

The measurement of morning SC at 8–9 AM is recommended as the first‐line test for AI screening [11]. However, in patients affected by an oncologic disease, the collection of time‐sensitive biochemical sample may be impaired by reduced adherence and/or logistic difficulties [7].

When the morning SC falls into the grey zone (ranging from 83 to 414 nmol/L), a dynamic assessment of HPA axis is unavoidable and, whichever test is used, peak stimulated SC ≤ 500 nmol/L is indicative of AI [11]. However, in the early phase of ICI‐related secondary AI, a rise in cortisol can be observed before adrenal atrophy has occurred; thus, a dynamic test may be falsely reassuring [12].

The thresholds for AI diagnosis or exclusion had been identified by dosing SC with outdated fluorometric assays. The risk of inappropriately labelled healthy individuals as subjects with AI is notable in an era of more specific immunoassay, particularly in case of liquid chromatography coupled with tandem mass spectrometry (LC–MS/MS). Recently, several studies have tried to identify assay‐specific cut‐offs to ensure a more accurate interpretation of SC [13, 14].

In the last years, alternative diagnostic tools to SC have been explored for identifying patients with AI, showing promising results. Debono et al. [15] found that home waking salivary cortisone sampling demonstrated similar accuracy to the ACTH stimulation test for diagnosing AI; additionally, patients preferred this approach over hospital‐based testing.

So far, different screening algorithms have been proposed for the detection of ICI‐related AI in the outpatient setting. However, these indications are mostly based on expert opinions and do not derive from studies involving ICI‐treated patients; the recommended thresholds are set to safeguard patient health by ensuring that treatment begins whenever cortisol deficiency is suspected, even in the absence of a definitive diagnosis of AI [4, 7, 16].

The main scope of our prospective observational study was to analyze the role of morning SC in the diagnosis of ICI‐induced AI in ICI‐treated patient, with the aim of reducing the grey zone and limiting the need for dynamic assessment of HPA axis. We also explored the potential contribution of salivary cortisol and cortisone dosage upon awakening in this setting.

## 2 Patients and Methods

We evaluated all adult ICI‐treated cancer patients followed‐up at the Oncologic Endocrinology Unit of ‘Città della Salute e della Scienza Hospital’ in Turin between May 2023 and August 2024, whose morning SC (measured at 8:00–9:00 AM) fell into the grey zone identified by the Endocrine Society (ES) guidelines (83–414 nmol/L) in two occasions [11]. We excluded from the study subjects on current or recently discontinued glucocorticoid‐based therapy (within 1 month; 3 months in case of intramuscular/intra‐articular formulation), except patients assuming adrenal replacement therapy at physiological doses; in those cases, morning SC was measured after 24‐h interruption of hormone replacement. We also excluded women on oral oestrogen therapy, patients with acute clinical conditions, individuals on working night shifts, and subjects unable to produce a suitable saliva sample for analysis.

A dynamic evaluation of the HPA axis was performed by low‐dose ACTH stimulation test (1 µg intravenous cosyntropin); the test started after an overnight fast between 8:00 and 9:00 AM and SC was measured at baseline, after 30 and 60 min.

To limit potential confounders, patients on adrenal replacement therapy or assuming benzodiazepine therapy were asked to refrain from taking the drug the day before the test and to assume their daily dose after the test; subjects on opioid therapy were asked to assume their daily dose after the test.

Additional blood analytes were evaluated: glucose, sodium, potassium, albumin, ACTH, thyroid‐stimulating hormone and free thyroid fractions (in patients not assuming l‐thyroxine therapy for primary hypothyroidism), follicle stimulating hormone (FSH) and luteinizing hormone (LH) in postmenopausal women (along with estradiol in premenopausal women), LH along with testosterone and sex hormone binding globulin (SHBG) in men.

On the same morning of the dynamic evaluation, patients were asked to collect at home a saliva sample upon awakening for salivary cortisol and cortisone assessment, after having received proper instruction and a tube containing a cotton swab, certified for saliva sample collection intended for cortisol and cortisone measurement (tube name: *Salivette*, manufactured by Sarstedt).

All biochemical and hormonal determinations were performed at our hospital laboratory. SC measurement (1 μg/L = 2.76 nmol/L) was conducted using the Abbott Alinity i Cortisol kit, a chemiluminescent microparticle immunoassay, with a lower limit of quantification of 76.1 nmol/L. Salivary cortisol (1 ng/dL = (nmol/L/27.8) × 1000) and cortisone (1 ng/dL = (nmol/L/27.6) × 1000) determinations were performed using the Salivary Cortisol and Cortisone LC–MS/MS kit (manufactured by Chromsystems Instrumental and Chemicals GmbH), with a lower quantification limit of 0.47 nmol/L for cortisol and 2 nmol/L for cortisone.

Demographic, anthropometric and relevant clinical data were collected on the occasion of the ACTH stimulation test, assessing adverse events (AEs) by Common Terminology Criteria for Adverse Events (CTCAE) Version 5.0 [17]. All subjects provided informed consent to participate in the study, which was approved by the Local Ethics Committee (Code 00096/2022).

### 2.1 Statistical Analysis

Continuous variables were reported as mean and standard deviation (SD) or median and interquartile range (IQR), while categorical variables were presented as frequencies (*N* and %). The normal distribution of continuous variables was assessed using the Kolmogorov–Smirnov test. The Mann–Whitney test was used to compare medians of continuous variables. The association between the morning SC measured before the ACTH test and the peak stimulated SC was evaluated using Pearson's correlation coefficient (*r*); the same analysis was performed to explore the association between salivary cortisol/cortisone upon wakening and peak stimulated SC.

To interpret the results of the ACTH test, it was employed both the threshold provided by ES guidelines [11] and that suggested by Lazarus and colleagues for the assay used in our laboratory [14]. Based on these two cut‐offs for peak stimulated SC, two different ROC curves were elaborated to assess the diagnostic performance of morning SC measured before the ACTH test in predicting the outcome of the ACTH stimulation test. The ROC curves show sensitivity versus (1 − specificity) for different morning SC cut‐off points. For each of the two curves, the area under the ROC curve (AUC) was calculated, representing the overall diagnostic performance of morning SC measurement; higher AUC values indicate better overall test performance.

Aiming to narrow the grey zone defined by the ES guidelines [11], through the analysis of the ROC curves, we identified the best morning SC values able to predict or to exclude a pathological response of the dynamic evaluation. For each ROC curve, we determined the morning SC values within the grey zone that identified with the highest specificity or the highest sensitivity the patients who should not undergo the ACTH stimulation test, because the response would be certainly pathological or physiological, respectively. For each of these thresholds, sensitivity, specificity, positive predictive value (PPV), negative predictive value (NPV), positive likelihood ratio (LR+), and negative likelihood ratio (LR−) were reported.

A significance level of *p* < 0.05 was considered statistically significant. All analyses were performed using the software ‘Stata 15.1’ (StataCorp LP, College Station, TX, USA).

## 3 Results

The sample included 30 subjects treated with ICI. Relevant data about demographics, clinical and therapeutic features are reported in Table 1.

**Table 1:** Relevant patients' data at the time of the ACTH stimulation test.

Age (years)—median (IQR)	64.9 (53.7–74.9)
Sex—*N* (%)	
Men	15 (50%)
Women	15 (50%)
Time from the start of therapy (months)—median (IQR)	10.6 (7.6–25.2)
Neoplastic disease—*N* (%)	
Lung	7 (23.3%)
Melanoma	14 (46.7%)
Other	9 (30%)
Type of ICI—*N* (%)	
Anti PD‐1	24 (80%)
Anti PD‐L1	4 (13.3%)
Anti CTLA‐4 + anti PD‐1	2 (6.7%)
ART before the dynamic evaluation—*N* (%)	
Every day	10 (33.3%)
Only in case of stress	13 (43.3%)
Asthenia—*N* (%)	
Grade 1	11 (36.7%)
Grade 2	6 (20.0%)
Hypotension—*N* (%)	1 (3.3%)
Body mass index (Kg/m^2^)—*N* (%)	
< 18.5	—
> 25	19 (63.3%)
Hyponatraemia—*N* (%)	3 (10.0%)
Hyperkalaemia—*N* (%)	—
Hypoglycaemia—*N* (%)	—
Secondary hypothyroidism—*N* (%)	—
Secondary hypogonadism—*N* (%)	5 (16.7%)
Stomatitis—*N* (%)	
Grade 1	1 (3.3%)
Smoke—*N* (%)	2 (6.7%)
Opioid therapy—*N* (%)	2 (6.7%)
Benzodiazepine therapy—*N* (%)	3 (10.0%)
Hypoalbuminaemia—*N* (%)	1 (3.3%)
Severe liver impairment—*N* (%)	—

Abbreviations: AI, adrenal insufficiency; ART, adrenal replacement therapy; CTLA‐4, cytotoxic T‐lymphocyte‐associated protein‐4; ICI, immune‐checkpoint inhibitor; PD‐1, programmed death receptor 1; PD‐L1, programmed death ligand 1.

**Figure 1:**
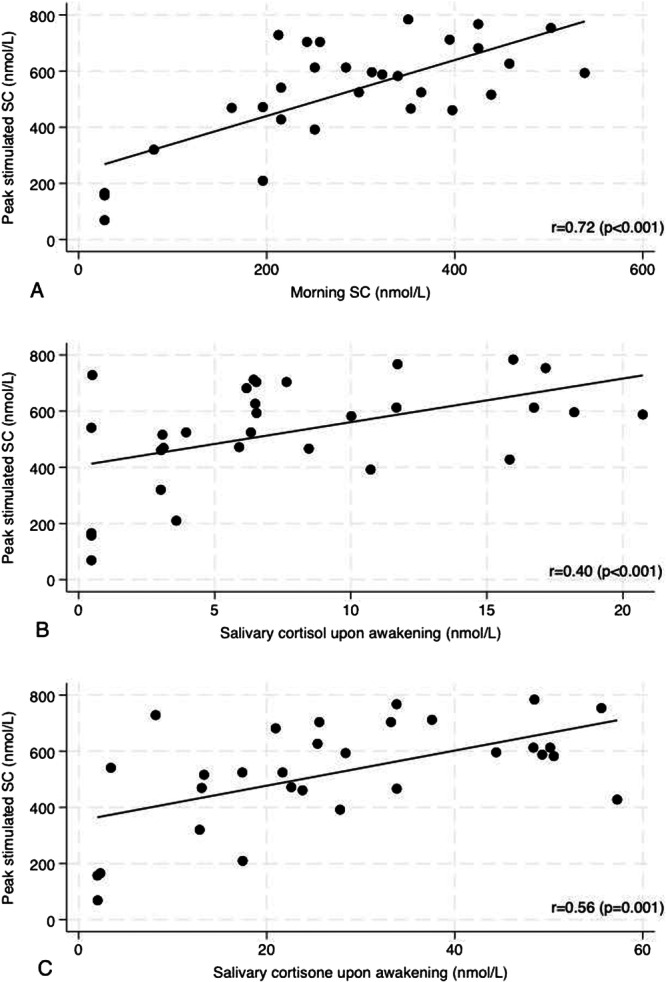
Correlation between morning SC and peak stimulated SC (A), salivary cortisol upon awakening and peak stimulated SC (B), salivary cortisone upon awakening and peak stimulated SC (C). SC, serum cortisol.

A good positive correlation was observed between the morning SC measured before the ACTH test and the peak stimulated SC (*r* = 0.72, *p* < 0.001), as displayed in Figure 1. The dynamic evaluation was well tolerated in all patients; the highest stimulated SC was reached at 30 min in 50% of cases.

Following the ES guidelines, on the sole basis of the morning SC re‐tested before the ACTH test, four cases would have been considered pathological, while hypocortisolism would have been ruled out in six patients; in all those cases, the diagnosis or the exclusion of AI was confirmed by the dynamic evaluation. Overall, by applying the cut‐off provided for peak stimulated SC by ES guidelines, AI was diagnosed in 36.7% of our sample. Lowering such threshold as suggested by Lazarus et al. [14], only 20% of patients showed a pathological response; therefore, 16.7% of the sample would have been reclassified with no need to hormone replacement.

**Figure 2:**
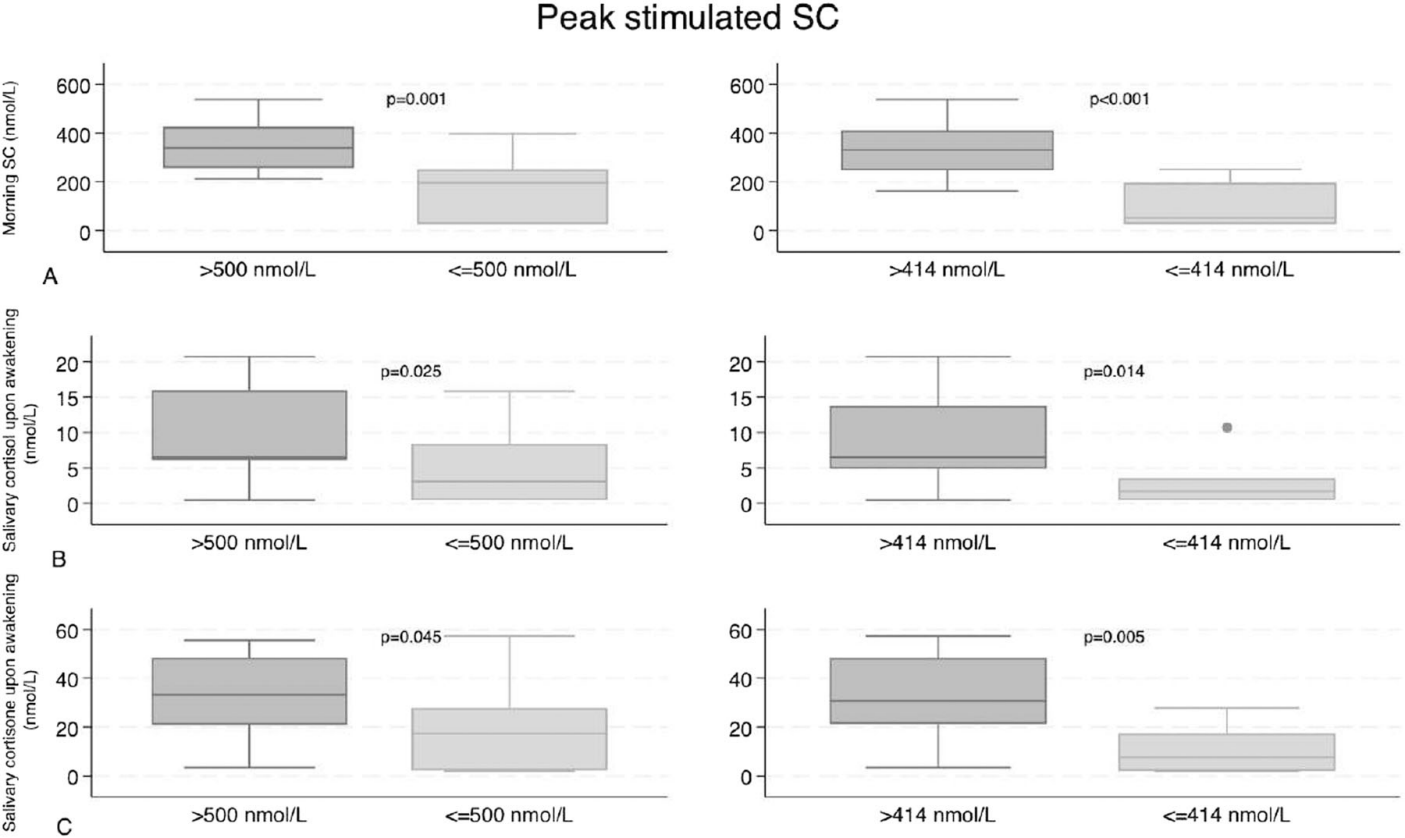
Distribution (median and IQR) of morning SC (A), salivary cortisol upon awakening (B), salivary cortisone upon awakening (C) in different subgroups: patients with peak stimulated SC ≤ 500 nmol/L and with peak stimulated SC > 500 nmol/L: patients with peak stimulated SC ≤ 414 nmol/L and with peak stimulated SC > 414 nmol/L. SC, serum cortisol.

The median morning SC measured before the ACTH test in the subgroups with and without AI (according to the two different cut‐offs for peak stimulated SC) were displayed in Figure 2.

All cases were central AI according to the ACTH levels; none of the subjects with AI displayed other pituitary hormonal impairment. Patients reporting asthenia and hyponatraemia were included both in the subgroup with and without AI. Applying the cut‐off value for stimulated SC of 500 and 414 nmol/L, 4 and 6 out of 10 patients with previous indication of assuming cortisone acetate daily were re‐classified as normocortisolemic subjects, respectively.

**Figure 3:**
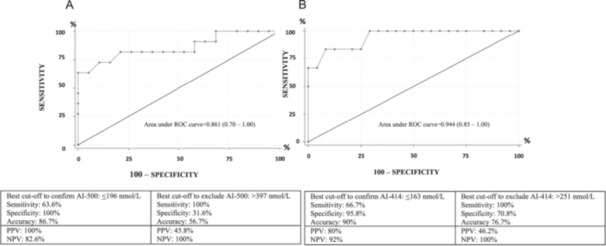
Diagnostic performance of morning SC in predicting the outcome of the ACTH stimulation test according to two ROC curve analyses elaborated based on different cut‐offs for peak stimulated SC. (A) AI defined by peak stimulated SC ≤ 500 nmol/L; (B) AI defined by peak stimulated SC ≤ 414 nmol/L. NPV, negative predictive value; PPV, positive predictive value; SC, serum cortisol.

According to the ROC curve analyses (Figure 3), a morning SC ≤ 196 nmol/L was the best value to predict AI according to the ES guidelines (sensitivity 63.6%, specificity 100%, accuracy 86.7%, PPV 100%, NPV 82.6%, LR+ 0, LR− 0.36), while the best value to exclude AI was > 397 nmol/L (sensitivity 100%, specificity 31.6%, accuracy 56.7%, PPV 45.8%, NPV 100%, LR+ 1.46, LR− 0). If we had decided to perform the ACTH stimulation test only in the patients with morning SC > 196 nmol/L but ≤ 397 nmol/L, only 17 subjects (56.7% of the sample) would have been tested; 4 of these cases (23.5%) would have been diagnosed with AI. Using these values to define the grey zone, no subject would have been classified as false positive or false negative.

A morning SC ≤ 163 nmol/L was the best value to predict AI according to the threshold for peak stimulated SC proposed by Lazarus et al. [14] (sensitivity 66.7%, specificity 95.8%, accuracy 90%, PPV 80%, NPV 92%, LR+ 16, LR− 0.35), while the best value to exclude AI was > 251 nmol/L (sensitivity 100%, specificity 70.8%, accuracy 76.7%, PPV 46.2%, NPV 100%, LR+ 3.42, LR− 0.29). If we had decided to perform the dynamic evaluation only in patients with morning SC > 163 nmol/L and ≤ 251 nmol/L, only 8 subjects (26.7%) would have been tested; 2 of these (25%) would have been diagnosed with AI. Using these values to define the grey zone, only a subject would have been classified as a false positive, without false negatives.

**Figure 4:**
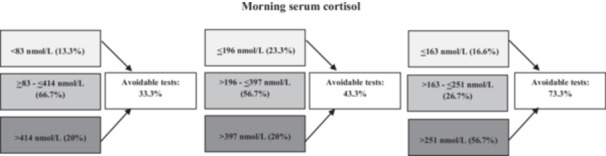
Percentage of avoidable ACTH stimulation tests on the basis of the morning SC in our population. We considered as the limits of the grey zone for morning SC both the thresholds defined by ES guidelines (≥ 83 and ≤ 414 nmol/L) and those derived from the analyses of the two ROC curves (> 196 and ≤ 397 nmol/L; > 163 nmol/L and ≤ 251 nmol/L). AI, adrenal insufficiency; SC, serum cortisol.

Figure 4 shows the number of potentially avoidable ACTH stimulation tests by the sole assessment of the morning SC, considering different limits for the definition of the grey zone.

Regarding the saliva samples collected upon awakening, a moderate correlation was found between salivary cortisol values and peak stimulated SC (*r* 0.40, *p* < 0.001) and between baseline salivary cortisone values and peak stimulated SC (*r* 0.56; *p* 0.001), as displayed in Figure 1. The median salivary cortisol and cortisone values at awakening in subgroups with and without AI according to the two different cut‐offs for peak stimulated SC are displayed in Figure 2.

## 4 Discussion

The number of patients exposed to ICI therapy is rapidly growing and ICI‐induced endocrinopathies are among the most frequently reported immune‐related AE.

An early recognition of ICI‐induced AI is essential, since the damage of HPA axis can potentially determine rapid‐onset and life‐threatening manifestations, including severe haemodynamic and electrolyte alterations; treatment should be started immediately if an adrenal crisis is suspected [16].

In our cohort, all cases of HPA dysfunction can be labelled as secondary AI, with low or inadequately normal ACTH levels. Hypocortisolism was not accompanied by secondary hypogonadism and/or central hypothyroidism and/or symptoms due to pituitary enlargement; all cases are likely to be IAD. This is not surprising if it is considered that many of them were treated with anti PD‐1 or anti PD‐L1. However, it must be underlined that the assessment of all pituitary hormones (e.g., somatotropic axis evaluation) and MRI were not routinely performed in all patients.

The clinical manifestations of AI in its milder form can easily be mistaken, overlapping with signs and symptoms due to other AEs of treatments or to the cancer itself (e.g., asthenia, poor oral intake, nausea and vomiting, hypotension) [7]. Fatigue was detected in more than half of our population; however, this symptom did not seem to be associated with ICI‐induced AI, even if the limited number of patients included in our study did not consent to draw definite conclusions. It also emerged that a relevant number of patients previously put on therapy with cortisone acetate showed an adequate response to ACTH stimulation test. In our real‐life practice, when morning SC falls within the grey zone, hormone replacement can be started to ensure patient safety if there is any possibility of cortisol deficiency based on the clinical evaluation. Even if a recovery of HPA axis cannot be excluded in some of our patients previously put on replacement therapy, it is also possible that the initial clinical evaluation was misleading in those subjects. Overall, our data support the indication of a routinary biochemical screening of HPA axis before the start of ICI and periodically during treatment, independently from the clinical assessment, as suggested in literature [6, 8, 9].

When morning SC levels are not suggestive for AI but at the same time do not allow ruling out a HPA axis alteration, ES guidelines recommend performing an additional dynamic testing. The insulin tolerance test (ITT) is traditionally considered the gold standard test for the dynamic evaluation of the HPA axis, assessing the SC response to insulin‐induced hypoglycaemia [11]. However, its use in the oncologic setting is limited due to its discomfort for patients, the need for close medical supervision, and contraindications in various conditions (e.g., health disease). A valuable alternative tool is represented by the ACTH stimulation test, performed either at standard‐dose (cosyntropin 250 µg) or low‐dose (cosyntropin 1 µg), which is less complex, safer and more tolerated than ITT, even if it still requires appropriate monitoring and multiple blood sampling [18]. However, in the early phase of ICI‐related secondary AI, the result of a dynamic test may be falsely reassuring, hence a single evaluation might not be conclusive [12]. Therefore, the diagnosis of AI has to largely rely on the measurement of morning SC in this setting [4].

In our population, the sole repetition of the morning SC measured before the ACTH test allowed a direct diagnosis or exclusion of AI in 33% of subjects; in those cases, the ACTH stimulation test would have been avoidable. Moreover, median values of morning SC in AI patients were significantly lower than normocortisolemic subjects and our analysis confirms a good positive linear correlation between morning SC levels and peak stimulated SC, in line with data from literature. Overall, our data suggested that morning SC could be considered a reliable tool for the screening of ICI‐related AI; in patients with non‐conclusive morning SC levels, a basal re‐evaluation of SC following its trend over time seems a possible alternative to an immediate dynamic assessment of the HPA axis.

On the basis on the ROC curve analyses, we identified thresholds for morning SC which would narrow the grey zone and consequently decrease the number of patients needing a dynamic evaluation, with significant clinical implications.

To interpret the results of ACTH test, we employed both the threshold provided by ES guidelines and an assay‐specific cut‐off for Abbott platform recently proposed in literature.

In its research, Lazarus and colleagues retrospectively analyzed 300 SC responses to ITT using the Abbott Architect and Alinity analyzer platforms in patients with suspected AI: the application a lower peak stimulated SC threshold of 414 nmol/L maintained a sensitivity of 100.0% and improved the specificity to 86.7%, avoiding in healthy patients a misdiagnosis of AI and an unnecessary replacement therapy with lifelong follow‐up [14]. In our population, 16.7% patients would not be labelled as AI subjects, by applying the new assay‐specific cut‐off rather than the traditional threshold.

It has to be considered that Lazarus and colleagues identified AI patients on the basis of a clinical outcome, after a comprehensive clinical review of electronic patient records. Differently from that study, in our population we could not use the clinical evaluation as a goal standard to assess a HPA deficiency, due to the overlap of symptoms characterizing the initial presentation of AI and those observed in cancer patients on antitumor treatments, also in case of adequate levels of SC. In this setting, the stimulated biochemical evaluation remains the most reliable tool to categorize patients. Our study was not designed to determine if our patients were healthy individuals who had been inappropriately considered affected by AI or subjects with AI missed by the new proposed cut‐off. We just wanted to explore the potential impact a recently proposed threshold for stimulated SC in the classification of patients, confirming the need for further analysis to investigate assay‐specific SC cut‐offs both able to detect AI and avoid unnecessary replacement therapy.

The use of the ITT test is extremely limited in the oncologic setting, mainly due to the discomfort for patients. However, we hypothesized that the results of the ACTH stimulation tests performed in our patients can be comparable to the results analysed by Lazarus and colleagues, since the interpretation of ITT and ACTH stimulation test is based on the same criteria according to ES guidelines [6].

It is well known that SC must be collected within an appropriate window because of the diurnal pattern of endogenous cortisol secretion; however, this indication adds complexity to the cancer patient's route, which is characterized by a high number of hospital accesses and tests. According to a recent study exploring the HPA screening practices during ICI at a tertiary‐care institution, 60% of SC tests were performed outside of the accepted time frame [7]. In our study, we explored the contribution of salivary cortisol and cortisone dosage upon awakening in this setting, as previously suggested for AI screening in literature [15]. In our laboratory, LC–MS/MS was employed for the determination of such salivary hormonal levels. Median values were lower in AI patients in comparison with NO AI subjects. A positive correlation was found between salivary cortisol or cortisone and peak stimulated SC; however, it must be underlined that the strength of the correlation between morning SC and peak stimulated SC was higher than those observed between these salivary hormones and peak stimulated SC.

While the prospective nature of this study is a strength, the limited sample represents a limitation of our preliminary research. Nevertheless, strict inclusion and exclusion criteria were employed to limit confounding factors and increase the quality of evidence on an issue where precise indications supported by solid data are lacking. Conditions altering the detection of cortisol levels in serum or saliva were registered in a very limited number of cases. Moreover, all hormonal determinations were homogeneously performed in the same laboratory using specific assays; clearly, the results may not be generalizable to other populations if different laboratory methods are used.

In conclusion, morning SC can be considered a reliable tool for the screening of ICI‐related AI among asymptomatic or mildly symptomatic subjects, whereas the sole clinical judgement may be misleading. The number of cases needing a dynamic stimulation test can be considerably reduced by the re‐evaluation of morning SC over time and the application of assay‐specific cut‐offs which consent to narrow the grey zone. This approach would reduce the complexity of the diagnostic pathway of ICI‐treated cancer patients and decrease healthcare‐related costs. The collection of salivary samples upon awakening for cortisol and cortisone dosage represents a promising tool for ICI‐related AI screening in selected cases, when patients cannot perform a morning hospital‐based test. Further investigations involving a higher number of patients are needed to validate these preliminary data.

## Conflicts of Interest

The authors declare no conflicts of interest.
